# Supplementary material for: Taming numerical errors in simulations of continuous variable non-Gaussian state preparation
Source: Sci Rep. 2022 Oct 4;12:16574. doi: 10.1038/s41598-022-19506-9 (PMC9532453; doi:10.1038/s41598-022-19506-9)
Supplement: Supplementary file 1 — Supplementary Information. [file 41598_2022_19506_MOESM1_ESM.pdf]

Taming numerical errors in simulations of continuous variable non-Gaussian state preparation

Jan Provazník[1,*], **Radim Filip**[1], **and Petr Marek**[1]

[1]Department of Optics, Palacký University, 17. listopadu 1192/12, 771 46 Olomouc, Czech Republic
[*]provaznik@optics.upol.cz

ABSTRACT

Numerical simulation of continuous variable quantum state preparation is a necessary tool for optimization of existing quantum information processing protocols. A powerful instrument for such simulation is the numerical computation in the Fock state representation. It unavoidably uses an approximation of the infinite-dimensional Fock space by finite complex vector spaces implementable with classical digital computers. In this approximation we analyze the accuracy of several currently available methods for computation of the truncated coherent displacement operator. To overcome their limitations we propose an alternative with improved accuracy based on the standard matrix exponential. We then employ the method in analysis of non-Gaussian state preparation scheme based on coherent displacement of a two mode squeezed vacuum with subsequent photon counting measurement. We compare different detection mechanisms, including avalanche photodiodes, their cascades, and photon number resolving detectors in the context of engineering non-linearly squeezed cubic states and construction of qubit-like superpositions between vacuum and single photon states.

Supplementary material

S.1 Ill-defined behavior of the displacement matrix

We introduced the closed form formula[1] for the displacement operator in Section 2 of the article and briefly discussed the shortcomings of its numerical realization using floating point arithmetic. In this section we discuss the shortcomings of its straightforward realization. The formula[1] is repeated here for clarity. Each matrix element of the $\hat{D}(\xi)$ matrix is given by

$$\langle m|\hat{D}(\xi)|n\rangle = \sqrt{\frac{n!}{m!}}\xi^{m-n}\exp\left(-\frac{1}{2}|\xi|^2\right)L_n^{(m-n)}(|\xi|^2)\,, \quad m \geq n \tag{S.1}$$

where $L_\beta^\alpha(x)$ denotes an associated Laguerre polynomial function[2]. A straightforward numerical implementation of (S.1) suffers from limitations of FP arithmetic. If implemented exactly as it stands in (S.1), the first term underflows for comparatively large m, while the second term overflows for $|\xi| > 1$ and large enough difference $m-n$. When both the numerical underflow and the overflow coincide, the ill-defined expression $0\times\infty$ is evaluated, resulting in error.

The computation of (S.1) can be simplified by replacing the fraction in the first term with

$$\frac{1}{n+1}\frac{1}{n+2}\cdots\frac{1}{m-1}\frac{1}{m}, \quad \text{resp.} \quad \frac{1}{(n+1)(n+2)\cdots(m-1)m}\,. \tag{S.2}$$

Nevertheless, this substitution is not going to prevent the pathological $0\times\infty$ as expressions with either sufficiently high difference $m-n$ will still occur. This substitution also affects the numerical accuracy of (S.1). It furthermore depends on the way the substitution is evaluated. Multiplying individual fractions gives a different outcome than inverting the product as a whole.

One of the pathological occurrences of $0\times\infty$ can be observed for $\langle m|\hat{D}(\xi)|0\rangle$ with $|\xi| > 1$. The denominator in the substitution (S.2) reduces into $m!$ and the factorial overflows for sufficiently large m. This threshold can be estimated with the aid of the improved error bounds[3] for Stirling's approximation

$$\sqrt{2\pi}\exp\left[\ln(m)\left(m+\frac{1}{2}\right)-m+\frac{1}{12m+1}\right] < m! < \sqrt{2\pi}\exp\left[\ln(m)\left(m+\frac{1}{2}\right)-m+\frac{1}{12m}\right]\,. \tag{S.3}$$

We can exploit the upper bound to determine the largest safe value of m for which the factorial $m!$ does not overflow by comparing the bound with the largest representable FP number within the particular FP system used in calculation of (S.1).

Similarly we can determine the value of m for which $m!$ definitely overflows from the lower bound. We investigate the qualitative behaviour of the closed form formula (S.1) by studying a simplified model expression

$$\frac{|\xi|^m}{m!} \tag{S.4}$$

in the context of the standard double FP precision. This expression comprises the disputable components of the first two terms in the original formula and becomes **invalid** under the same circumstances. Within Fig. S.1 we identified four regions with qualitatively different outcomes for different combinations of the $|\xi|$ and m parameters. The *dashed horizontal* line determines the maximal m for which $m!$ does not overflow and the *solid* curve marks the maximal possible m such that $|\xi|^m$ does not overflow. These lines divide the area into four regions. In the *top-left back-slashed* region $m!$ overflows, while $|\xi|^m$ remains finite. Even though (S.4) is numerically valid in this region, its value might be incorrect as it always zeroes out due to the overflown denominator. Similarly the formula remains a valid, albeit incorrect, expression in the *central-right slashed* region where $m!$ is a regular number, however, $|\xi|^m$ overflows and therefore (S.4) reaches numerical infinity. In the *top* (unmarked) region we obtain the pathological $0 \times \infty$ since both $|\xi|^m$ and $m!$ overflow at the same time.

So far no discussed region provides satisfactory results. The formula (S.4) can only attain suitable numerical values within the *cross-stitched* region. While no overflows happen within this region we should still treat the resulting value with care as it might still succumb to loss of accuracy due to rounding errors since large numbers are generally involved in the computation. In essence this region only provides a necessary, rather than sufficient, condition for different combinations of the parameters $|\xi|$ and m.

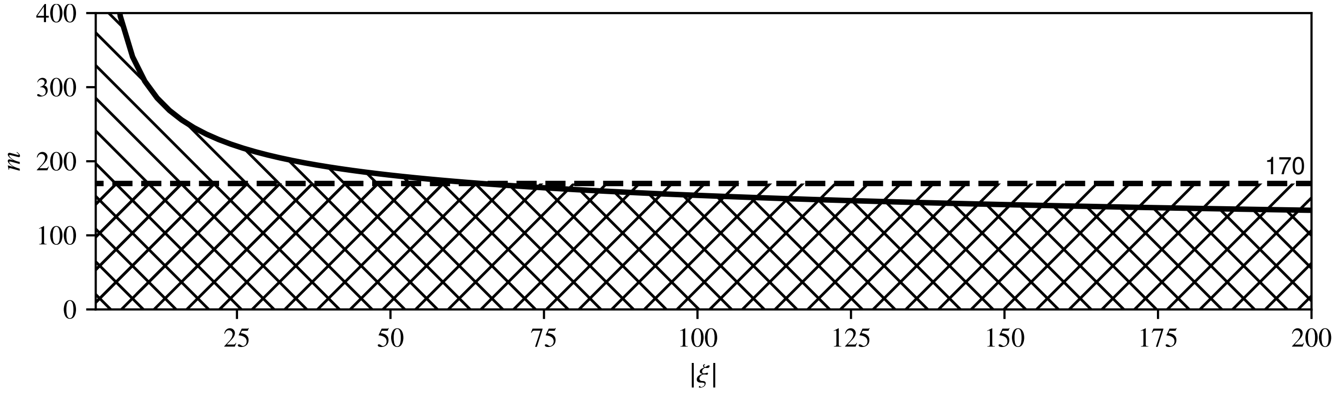

Figure S.1. Demonstration of the qualitative behavior of (S.1) on a surrogate model (S.4) with numerical limits of the standard double precision floating point arithmetic. The solid curve shows maximal m such that $|\xi|^m$ does not overflow; the dashed line shows maximal m such that $m!$ does not overflow. These two divide $|\xi|$ and m parameters into four distinct regions. We get the pathological $0 \times \infty$ expression in the top unmarked region. In the slashed region on the right, the expression diverges to infinity. In the left back-slashed region, the expression incorrectly zeroes out due to overflow in $m!$. Neither of these regions provides satisfactory values of (S.4) and consequently of the formula (S.1). Only the parameters from the bottom cross-stitched region lead to numerically valid, finite valued results.

In this discussion we established the viable range of parameters in the model (S.4). The first two terms in the closed form formula (S.1) lead to numerically valid expressions under the same circumstances. One of the implications is that (S.1) can not be used to construct the displacement matrix on arbitrarily large truncated Fock spaces. In practical applications, when utilizing double precision for the numerical simulation, the maximal attainable dimension is upper-bound by 170, regardless of the displacement modulus $|\xi|$.

S.2 Further comments regarding the displacement matrix

The straightforward realization of (S.1) we discussed in Section 1 is not the only conceivable approach. For the sake of completeness, let us briefly overview couple of the possibilities with focus solely on the first two terms.

In (S.2) we replaced the fraction in the first term with $[(n+1)(n+2)\cdots(m-1)\cdot m]^{-1}$. This simple substitution aims to improve the overall accuracy by avoiding the large numbers that would be otherwise produced the factorials. Note that the way this product is evaluated impacts its accuracy. Multiplying individual fractions yields different outcome is from inverting the product as a whole. Nevertheless, this substitution does not prevent the pathological $0 \times \infty$.

We could also replace the first two terms entirely with

$$\frac{\xi}{\sqrt{n+1}}\frac{\xi}{\sqrt{n+2}}\cdots\frac{\xi}{\sqrt{m}} \equiv \xi^{m-n}\sqrt{\frac{n!}{m!}}\,. \tag{S.5}$$

While this approach avoids the pathological $0 \times \infty$ product over a broader range of m, n and $|\xi|$ parameters, it also amplifies rounding errors with terms greater than 1.

References

1. Cahill, K. E. & Glauber, R. J. Density operators and quasiprobability distributions. *Phys. Rev.* **177**, 1882–1902, DOI: 10.1103/physrev.177.1882 (1969).

2. Bateman, H. *Higher transcendental functions* (R.E. Krieger Pub. Co, Malabar, Florida, 1981).

3. Robbins, H. A remark on stirling's formula. *The Am. Math. Mon.* **62**, 26, DOI: 10.2307/2308012 (1955).
